# *C9orf72* arginine‐rich dipeptide repeat proteins promote assembly and impair dynamics of processing body

**DOI:** 10.1002/alz70861_108268

**Published:** 2025-12-23

**Authors:** Yongjie Zhang, Yanwei Wu, Wei Shao, Tania F Gendron, Jorge Alaiz Noya, Caroline J. Jones, Karen Jansen‐West, Lillian Daughrity, Monica Castanedes‐Casey, Björn Oskarsson, Dennis W. Dickson, Leonard Petrucelli

**Affiliations:** ^1^ Mayo Clinic, Jacksonville, FL USA

## Abstract

**Background:**

G_4_C_2_ repeat expansions in *C9orf72* gene are the most common genetic cause of frontotemporal dementia (FTD) and amyotrophic lateral sclerosis (ALS). Mounting evidence indicates that dipeptide repeat (DPR) proteins translated from the expanded repeats, in particular poly(GR) and poly(PR), play a significant role. We and others have shown that these positively‐charged arginine‐rich DPR proteins induce the assembly of stress granules (SGs) and impair their disassembly. While distinct from SGs, processing bodies (PBs) are also cytoplasmic messenger ribonucleoprotein condensates that assemble upon stress. However, the impacts of arginine‐rich DPR proteins on PB assembly and dynamics have yet to be investigated.

**Method:**

We conducted immunofluorescence staining for arginine‐rich DPR proteins, SG‐ and PB‐associated markers in cultured cells and mouse brain. Additionally, we performed *in vitro* liquid‐liquid phase separation (LLPS) using recombinant proteins and assessed molecular dynamics of SG and PB through fluorescence recovery (FRAP) after photobleaching in living cells.

**Result:**

Here, we observed that both GFP‐(GR)_100_ and GFP‐(PR)_100_ formed cytosolic inclusions immunopositive PB proteins. Unlike GFP‐(GR)_100_ and GFP‐(PR)_100_ inclusions, cellular cytoplasmic GFP‐(GA)_100_ inclusions did not contain PB proteins. We subsequently examined whether the phenomena observed in cultured cells occur in vivo. We observed that PB proteins were recruited to poly(GR) inclusions in cells with aggregated poly(GR). Next, we examined the dynamics of poly(GR)‐induced PBs in cultured cells using the FRAP technique. We observed that the recovery of stress‐induced PBs was more rapid than that of PBs in HEK293T cells expressing GFP‐(GR)_100_. Lastly, mechanistic studies revealed that poly(GR) interacts with PB proteins to form condensates in vitro via LLPS.

**Conclusion:**

Our results provide both in vitro and in vivo evidence that arginine‐rich DPRs induce the spontaneous formation of PBs via LLPS. Our results also indicate that poly(GR)‐induced PBs are more stable than the transient and dynamic conventional PBs. Given that PBs uniquely enriched with mRNA degradation and decay factors, the consequences of impairing these processes are expected to contribute to abnormal RNA metabolism in c9FTD/ALS.

